# Retrorectal cystic hamartoma unmasked by coccygeal fracture: a case report and review of presacral tailgut cysts and surgical management

**DOI:** 10.1097/RC9.0000000000000095

**Published:** 2026-02-04

**Authors:** Kaiser O’Sahil Sadiq, Ashley Alden, Jin K. Kim, Nicole D. Riddle, Allen Paul Chudzinski

**Affiliations:** a Department of Surgery, Division of Colorectal Surgery, UC San Diego, San Diego, California, USA; b Department of Surgery, University of South Florida, Tampa, Florida, USA; c Department of Pathology, University of South Florida, Tampa, Florida, USA

**Keywords:** perianal tumor, perineal mass, presacral tumor, retrorectal cystic hamartoma, retrorectal tumor, tailgut cyst

## Abstract

**Introduction:**

Tailgut cysts, or retrorectal cystic hamartomas, are uncommon developmental lesions found in the theoretical space between the rectum and the sacrum. Frequently asymptomatic and incidentally detected, they occur predominantly in women. Compression of nearby structures may cause perineal and back pain and even obstructed labor.

**Case Report:**

We report a case of a 50-year-old man with prior coccygeal fracture who presented with dull perineal pain, worsened by prolonged sitting. MRI revealed a cystic mass, and surgical excision was performed via a posterior perineal approach. Postoperative recovery was uneventful apart from surgery-related increased perineal pain. Pathology demonstrated a retrorectal cystic hamartoma.

**Discussion:**

Tailgut cysts are thought to originate from the embryonic tail, which normally regresses by the eighth week of gestation. MRI is the preferred imaging modality, with cysts appearing hypointense on T1 and hyperintense on T2 images. Solid components or irregular cyst walls on imaging may suggest malignancy. Preoperative biopsy carries risks of hemorrhage, infection, and tract seeding. In purely cystic lesions, complete surgical resection without biopsy is preferred. A perineal approach is recommended for small lesions below S3, while abdominal and abdominoperineal approaches are needed for larger lesions and those superior to S3. Routine coccygectomy was previously thought necessary to prevent recurrence but is now understood to be avoidable unless needed for exposure or the cyst is adherent. Malignant transformation occurs in up to 27% of cases, with recurrence up to 16%. Complete excision is essential to prevent recurrence and obtain a definitive diagnosis.

**Conclusion:**

Tailgut cysts, the most common retrorectal tumors, are often asymptomatic. MRI is the preferred imaging modality. Complete surgical resection is crucial to prevent malignancy and recurrence. The role of preoperative biopsy is controversial, and routine coccygectomy is no longer recommended.

## Introduction

The incidence of tumors in the retrorectal space is estimated to be between 0.9 and 6.3 patients per year, accounting for 1 in 40 000 hospital admissions^[^1-4^]^. However, as most reports originate from tertiary referral centers, the true incidence may be difficult to determine[5]. Tumors in this region are broadly categorized into congenital, inflammatory, neurogenic, osseous, and miscellaneous types and are further subcategorized as benign or malignant[6]. Clinical manifestations vary widely depending on the underlying etiology. Tailgut cysts, also called retrorectal cystic hamartomas or mucin-secreting cysts, are the most common cause of retrorectal cystic lesions^[^1,4,7^]^. Symptoms may result from compression of nearby anatomy^[^8-10^]^, but over half are asymptomatic and incidentally detected^[^11,12^]^. The retrorectal space is a potential space representing the lower portion of the presacral space. It is defined anatomically by the lower sacrum and coccyx posteriorly, the rectum anteriorly, the ureters and iliac vessels laterally, the peritoneal reflection superiorly, and the levator ani and pelvic floor inferiorly^[^13,14^]^. Here, we present a case report and a review of the literature that outlines the management for masses found in this region. This case has been reported in line with the SCARE criteria[15].

## Case presentation

**Figure 1.**
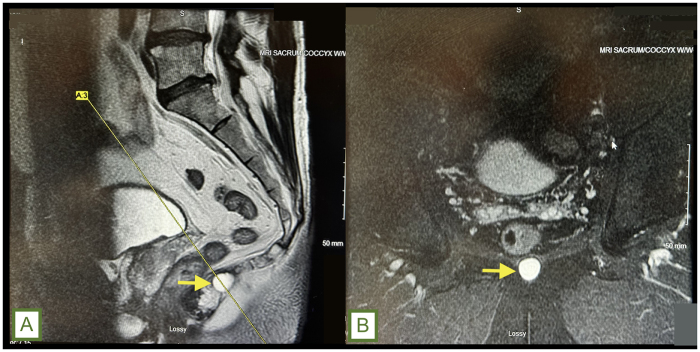
(A) Sagittal section of T2-weighted MRI and (B) coronal section T2 STIR MRI of the sacrum and coccyx showing a cyst in the retrorectal space (yellow arrow).

A 50-year-old otherwise healthy male presented to our hospital with complaints of dull, constant perineal pain, aggravated by sitting. Pertinent medical history includes a history of coccygeal trauma resulting in a nonoperative fracture that was complicated by a secondarily infected seroma requiring surgical drainage at an outside hospital. On exam, he was hemodynamically normal and non-septic appearing without a leukocytosis or other significant hematologic dyscrasias, but reported a lifestyle-limiting pain ranging between 2 and 5 out of 10. Further investigation with MRI of the sacrum and coccyx with and without contrast revealed a coccygeal cyst (Fig. 1A and B). Based on imaging, our presumed diagnosis was a presacral cystic mass. A subsequent colonoscopy was performed to further characterize this mass, which ruled out transmural invasion of the rectum. Given persistent pain, the patient opted for surgical intervention for definitive management.

**Figure 2.**
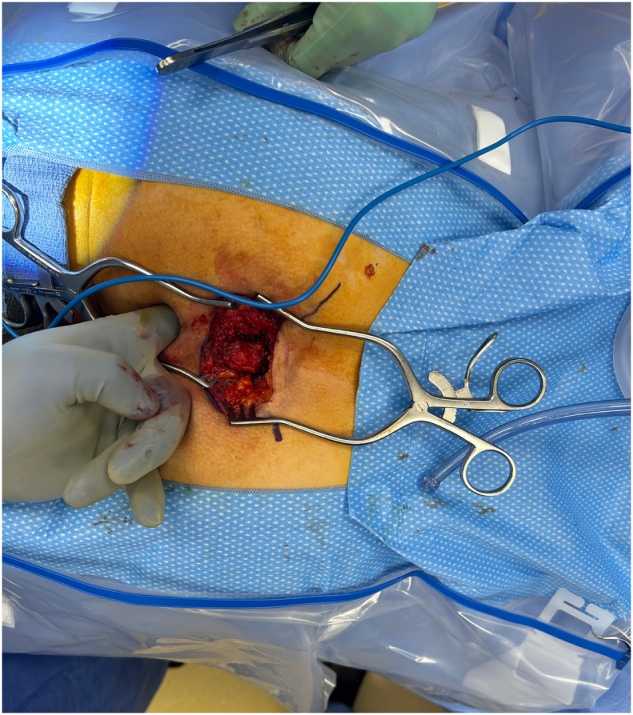
Intraoperative photograph showing digital rectal manipulation facilitating the dissection of retrorectal cyst.

**Figure 3.**
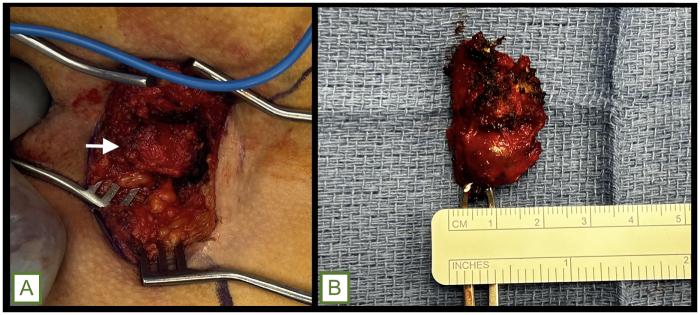
(A) Intraoperative photograph showing the dissected retrorectal mass (white arrow). (B) Photograph of the resected surgical specimen.

**Figure 4.**
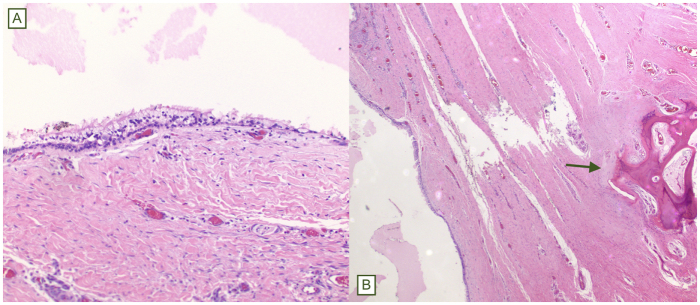
Histopathological slides showing (A) ciliated columnar epithelium with fibroconnective tissue on high power, and (B) proximity to bone (green arrow) on low power.

The patient was positioned in a prone jack-knife position with the buttocks taped apart to optimize exposure of the sacrococcygeal and perineal regions. Preoperative bowel preparation and prophylactic antibiotics were administered in accordance with institutional protocols, and the rectum was irrigated with a solution of povidone-iodine. A curvilinear S-shaped incision was made from above the tip of the coccyx toward the anus to expose the retrorectal space. Subcutaneous tissue was dissected, revealing an approximately 2 × 3 cm cystic lesion that was intimately associated with the rectal wall but nonadherent to adjacent structures. Meticulous circumferential dissection was required to separate the cystic mass from the posterior wall of the rectum without rupture. Digital rectal manipulation facilitated dissection by extruding the cyst toward the incision. By gently inserting a finger into the rectal lumen and applying controlled pressure, the cyst was extruded posteriorly toward the incision site. This maneuver significantly eased the dissection plane, reducing traction on the rectal wall and minimizing risk of inadvertent injury to the rectal wall (Fig. 2). Following complete mobilization, the cystic mass was excised en bloc, ensuring the entire capsule was removed intact (Fig. 3A and B). Hemostasis was achieved using electrocautery. After excision, the rectal lumen was irrigated once more with povidone-iodine solution. Proctoscopic evaluation was then performed to carefully inspect the rectal mucosa and muscular wall for any inadvertent perforation or injury. The integrity of the rectal wall was confirmed, obviating the need for further repair. The deep potential space was closed in multiple layers to obliterate dead space and reduce the risk of fluid accumulation, and the skin was closed with horizontal mattress sutures. A drain was left in the subcutaneous space for 7 days postoperatively to reduce seroma and potential abscess complications. Despite inadequate pain control in the immediate postoperative period and persistent watery stools due to the residual effects of bowel preparation, his postoperative course was uncomplicated. He was discharged the same day with additional pain medication. Histopathological examination demonstrated a benign cyst with ciliated columnar epithelium and surrounding fibroconnective tissue (Fig. 4A and B), consistent with a diagnosis of a retrorectal cystic hamartoma. No recurrence has been detected in the 10 months following excision.

**HIGHLIGHTS**

- Rare pathology: Reports a case of a tailgut cyst (retrorectal cystic hamartoma) in a 50-year-old male with prior coccygeal trauma.
- Diagnostic imaging: MRI revealed a T2-hyperintense cystic mass, confirming the lesion’s cystic nature and aiding surgical planning.
- Surgical management: Successful posterior perineal approach for complete excision, emphasizing meticulous dissection to avoid rupture and rectal injury.
- Pathological findings: Histopathology identified ciliated columnar epithelium, consistent with a benign tailgut cyst, reinforcing the importance of tissue diagnosis.
- Literature review: Summarizes etiology, malignant potential (up to 27%), and surgical approaches, advocating for tailored strategies based on lesion location; tailgut cysts are often asymptomatic but may become symptomatic due to trauma or infection; MRI is the gold standard for preoperative evaluation, while biopsy is discouraged due to infection/malignancy risks; and complete surgical resection is curative, with coccygectomy reserved for adherent cases.

## Discussion

### Etiology and histology

Tailgut cysts represent an uncommon clinical entity thought to arise from the persistence of the hindgut extension into the true embryonic tail, which normally involutes by the eighth week of gestation^[^16–19^]^. Histopathologically, tailgut cysts often contain mucinous material and may be lined by various epithelia: transitional urothelial-type, squamous, and columnar intestinal-type or respiratory-type with ciliated cells, as in our case^[^18,20,21^]^. The definition of tailgut cysts in the literature is somewhat nebulous. In the first large series, Hjermstad defined tailgut cysts as those lined at least in part by transitional or columnar epithelium without a well-defined muscular wall, myenteric plexus, or serosa (to exclude duplication cysts)[19]. Ciliated epithelium is present in the embryonic gastrointestinal tract and is not limited solely to respiratory tissue[22]. Simple or pseudo-stratified ciliated columnar epithelium is present in half of the tailgut cysts[19].

### Clinical presentation

Over half of tailgut cysts may be asymptomatic and detected incidentally^[^11,12^]^. Symptoms, when present, may arise from infection or compression of adjacent structures, leading to perineal, low back, lower extremity, or buttock pain, as well as incontinence, constipation, sexual dysfunction, and even obstructed labor^[^8-10^]^. Retrorectal masses are more frequently detected in women of childbearing age, possibly due to an increased number of pelvic exams[8]. A mass may be palpable on digital rectal examination, and the presence of postanal dimpling may be confused with pilonidal pits or fistulae^[^2,5,8,23,24^]^. This funnel-shaped dimpling may be related to the filum terminale[25], and its presence is often associated with an underlying tailgut cyst^[^6,25–27^]^. Invasion of sacral nerves may produce perianal anesthesia and laxity of the anal sphincter[8], and infection may lead to recurrent perianal sepsis if misdiagnosed[28]. In our case, we hypothesize that enlargement of a pre-existing tailgut cyst occurred secondary to the known history of pelvic trauma and subsequent hemorrhage or infection, ultimately resulting in symptomatic nerve compression.

### Preoperative workup and diagnosis

MRI is the preferred imaging modality, with lesions usually appearing hypointense on T1-weighted images and hyperintense on T2-weighted images^[^29–31^]^. In contrast to these uncomplicated cysts, complicated cysts may be hyperintense on T1-weighted MRI due to hemorrhage, proteinaceous, or mucinous material contained within or may display intermediate T1 and T2 intensity with irregular cyst walls^[^30,32^]^. Tailgut cysts may be unilocular but are more frequently multilocular. A honeycomb pattern of small cysts surrounding a dominant cyst has also been described[33]. Imaging findings concerning malignancy include solid components and irregular cyst wall thickening.

Purely cystic lesions are rarely malignant, and therefore preoperative biopsy is not recommended as it can lead to a pelvic abscess by seeding bacteria or hemorrhage^[^2,14,27,28^]^. Preoperative biopsy is only beneficial for select pathologies where neoadjuvant therapy is indicated^[^7,10^]^. A thickened, irregular cyst wall may also be the result of secondary inflammation and not necessarily indicate underlying malignancy[20]. In case of confirmed malignancy, biopsy tracts must be resected en bloc, which may require the removal of an otherwise uninvolved rectum or vagina[10]. Therefore, a CT-guided presacral extrarectal biopsy is preferred when necessary to minimize the risk of infection, perioperative complications, and local recurrence[8]. In a series of 21 retrorectal tumors, 47.6% of cases underwent biopsy, with only 1 case (4.8%) managed nonoperatively after detecting metastatic choroidal melanoma[34].

Endoscopy may be useful in defining the proximal extent of the lesion and ruling out rectal involvement[10], while endorectal ultrasound can assist in determining the extent of dissection required[24]. In the largest series to date, 45% of tailgut cysts were definitively diagnosed radiologically[35].

### Sequelae and complications

The reported risk of malignancy varies between 0 and 27%, with pathologies including adenocarcinoma, carcinoid, sarcoma, neuroendocrine tumors, and squamous cell carcinomas^[^8,14,19,24,28,33,36–39^]^. This is likely a result of the totipotent embryological cell lineages present in the cyst[35]. Additionally, 50% of malignant cysts may not be suspected to be malignant preoperatively[20]. Reported literature likely overestimates the risk of malignant transformation; however, the largest series from the Mayo Clinic demonstrated the risk to be 10%[35]. Serial imaging with MRI may be indicated when surgery is relatively contraindicated to surveil the development of a solid component or wall thickening to detect potential malignancy[7]. Recurrence rates vary from 0 to 16% with follow-up up to 60 months, often resulting from incomplete resection^[^12,14,19,35–37,40,41^]^.

### Surgical planning

The choice of surgical approach for presacral masses is determined by several factors, including the size, location, and nature of the lesion, as well as its relationship to adjacent structures such as the sacrum and rectum.

A perineal approach is recommended for small lesions below the level of S3, which can be performed transsacrally, transsacrococcygeally, transrectally, transanorectally, and transsphinterically. A prone jack-knife position with buttocks taped apart or a dorsal lithotomy position may be used, and a linear horizontal, longitudinal, radial, V-shaped, or curvilinear incision may be used. Transection of the anococcygeal ligament and levator ani, disarticulation of the coccyx, detachment of the gluteus maximus, and distal sacrectomy may be required in a transsacrococcygeal approach[8]. The intersphincteric approach for low-lying, small, and benign lesions involves blunt dissection of the internal sphincter up to the puborectalis, developing the intersphincteric plane, and entering the presacral space superiorly^[^10,24^]^. The perineal approach may postoperatively lead to seroma, infection, and lower limb weakness[12]. The posterior approach was used in 77% of tailgut cysts in the largest single-institution series[35].

Routine resection of the coccyx was once thought to be necessary to remove a potential nidus of totipotent cells, thus avoiding recurrence[28]. This is no longer recommended unless the cyst is adherent or coccygectomy is needed for adequate exposure. Recent series have reported coccygectomy rates ranging between 16.7 and 76.4%^[^12,28^]^.

A transabdominal approach is recommended for lesions without sacral involvement that do not extend below S4[10]. Compared to an open approach, laparoscopic dissection of the rectum away from the anterior surface of the lesion and dissection of the posterior surface away from the presacral fascia provides better visualization^[^42–44^]^. The middle sacral artery may need to be ligated[10].

Lesions extending above and below S3 necessitate a combined abdominoperineal approach in a modified dorsal lithotomy position^[^10,13,41^]^. The sigmoid is mobilized through a low midline incision, and the mesorectum is dissected from the presacral fascia below the sacral promontory until the lesion is reached. Isolated lesions are dissected circumferentially, but the involvement of adjacent structures may require en bloc resection. Preserving at least one S3 nerve root maintains urinary and fecal continence; therefore, the transection of both roots necessitates the formation of an end colostomy. Dural tears should be identified and repaired, and drains and musculocutaneous flaps may be required[10].

Robotic-assisted trans-abdominal approach has also been recently described. The enhanced visualization and articulation of robotic platforms may allow the resection of tumors below S3 with a trans-abdominal anterior-only approach and subsequent extraction through a Pfannensteil incision, potentially decreasing the morbidity associated with a perineal approach[45]. The perineal portion of the procedure is then performed as described previously. The sacrospinous and sacrotuberous ligaments are divided along with the piriformis to expose the sciatic nerves before an osteotomy and en bloc resection. Routine mechanical and antibiotic preparation is indicated in all patients if rectal resection or closure is necessary[8].

## Conclusions

Tailgut cysts are uncommon but represent the most common etiology of retrorectal tumors. Frequently asymptomatic, MRI is the imaging modality of choice. Owing to the risk of malignancy and recurrence, complete surgical resection is essential for tissue diagnosis and definitive treatment. The role of preoperative biopsy is controversial, and routine coccygectomy is no longer recommended.

## Research registration unique identifying number (UIN)

This is a case report and was not registered. It was accepted after undergoing peer review. No datasets were generated as this is a case report of a single patient.
